# Potential application of traditional Chinese medicine in cerebral ischemia—Focusing on ferroptosis

**DOI:** 10.3389/fphar.2022.963179

**Published:** 2022-09-23

**Authors:** Fengyan Zhao, Caiwang Peng, Yang Sun, Hengli Li, Ke Du, Fang Liu

**Affiliations:** ^1^ School of Pharmacy, Hunan University of Chinese Medicine, Changsha, China; ^2^ Center for Standardization and Functional Engineering of Traditional Chinese Medicine in Hunan Province, Changsha, China; ^3^ Key Laboratory of Modern Research of TCM, Education Department of Hunan Province, Changsha, China

**Keywords:** traditional Chinese medicine(TCM), cerebral ischemia, ferroptosis, apoptosis, autophagy

## Abstract

Traditional Chinese medicine (TCM) has attracted a great deal of attention in the treatment of cerebral ischemia is credited with the remarkable neuroprotective effects. However, the imperfect functional mechanism of TCM is a major obstacle to their application. Many studies have been conducted to illustrate the pathophysiology of post-ischemic cerebral ischemia by elucidating the neuronal cell death pathway. Meanwhile, a new type of cell death, ferroptosis, is gradually being recognized in various diseases and is becoming a new pathway of therapeutic intervention strategy to solve many health problems. Especially since ferroptosis has been found to be closely involved into the pathogenesis of cerebral ischemia, it has been considered as a key target in the treatment of cerebral ischemia. Therefore, this paper reviews the latest research findings about the treatment of cerebral ischemia with TCM focused on ferroptosis as a target. Also, in order to explores the possibility of a new approach to treat cerebral ischemia with TCM, we discusses the correlation between ferroptosis and other cell death pathways such as apoptosis and autophagy, which would provide references for the following researches.

## Introduction

Cerebral ischemia is a major cerebrovascular disease with high disability and mortality rates, public health and life safety are seriously compromised (Feigin et al., 2015). During the course of cerebral ischemia, a series pathological reactions are triggered, including oxidative stress (Meyers et al., 2012), inflammatory response (Krishnamurthi et al., 2013), excitatory amino acid toxicity (Brott and Bogousslavsky, 2000) and calcium excess (Adams et al., 2007), leading to widespread neuronal death and neurological damage ultimately. Therefore, protecting neurons from damage becomes a neuroprotective strategy (Moskowitz et al., 2010). Currently, multiple programmed cell death (PCD) have been identified to be involved into neuronal death, and it is possible to protect against cerebral ischemic injury by inhibiting PCD-related signaling pathways (Hribljan et al., 2019; Radak et al., 2017; Yu et al., 2019; Liu et al., 2020). It has been established that ferroptosis is a new type of PCD, and the inhibition of ferroptosis could reduce cerebral ischemic injury (Yang et al., 2020; Speer et al., 2013; Li et al., 2018). Naturally, ferroptosis is considered to be a critical therapeutic target for cerebral ischemia.

In TCM theory, Qi-deficiency along with blood-stasis syndrome are the main pathogenesis of cerebral ischemia that is treated by invigorating Qi and removing blood circulation. The classic TCM formula commonly used in cerebral ischemia treatment, such as Buyang Huanwu Decoction (Chen et al., 2020), QiShenYiQi (Wang et al., 2020) and Xinglou Chengqi Decoction (Chen et al., 2017), all conform to the treatment characteristics. With increasing studies on the anti-cerebral ischemia effect of TCM, the neuroprotective effects of TCM have attracted much attention benefit from the remarkable neuroprotective effects (Xia et al., 2014; Tian et al., 2019), so several studies have try to revealed the mechanism of TCM to interven incerebral ischemia *via* ferroptosis.

This review focuses on ferroptosis, summarizes the existing TCM literature on the regulation of cerebral ischemia by targeting the ferroptosis pathway, and discusses its relation to other cell death pathway. In particular, the importance of ferroptosis in mediating cerebral ischemia is emphasized to further explore specific targets of cerebral ischemia by TCM, which would serve as a reference for potential applications of TCM.

## Relevant mechanisms of ferroptosis in cerebral ischemia

Ferroptosis is defined as iron-dependent regulatory programmed cell death caused by decreased activity of glutathione peroxidase 4 (GPX4), accumulation of reactive oxygen species (ROS) and lipid peroxides (Yang and Stockwell, 2015). Iron deposition in the basal ganglia, globus pallidus and white matter regions during critical ischemia/anoxic injury and subsequent resuscitation was reported as early as 1988 (Dietrich and Bradley, 1988). In addition, typical ferroptosis phenomena such as the increased lipid peroxide and the decreased glutathione (GSH) levels were observed after middle cerebral artery occlusion (MCAO) in mice (Ahmad et al., 2014). Furthermore, ferroptosis inhibitors (liproxstatin-1 or ferrostatin-1) were shown to effectively inhibit ischemia-reperfusion injury (Tuo et al., 2017). Thus, targeting ferroptosis have been proven to be an essential neuroprotective strategy against cerebral ischemia.

Up to date, it is widely recognized that there are three related mechanisms for the control of cerebral ischemia by targeting ferroptosis as follows. 1) Iron dyhomeostasis. During cerebral ischemia, large amounts of iron are released from transferrin (Lipscomb et al., 1998), inducing iron, transferrin and transferrin receptor (TFR) accumulates intracellularly (Park et al., 2011; DeGregorio-Rocasolano et al., 2019). Clinical studies have shown that increased hepcidin expression downregulates the iron efflux in brain cells by inducing ferroportin1 (FPN1), leading to iron overload and ferroptosis (Kong et al., 2019; Petrova et al., 2016). 2) Accumulation of lipid peroxide and ROS. *In vitro* model of cerebral ischemia, increases in acyl-coa synthetase long-chain family member 4 (ACSL4) and 12/15-lipoxygenase (12/15-LOX) have been shown to increase polyunsaturated fatty acids (PUFAs) such as arachidonic acid (AA) and adrenal acid (AdA). It is believed to promote the expression of PUFAs, which in turn stimulates lipid peroxidation and neuronal cell death (Yigitkanli et al., 2017; Cui et al., 2021). In addition, thrombin, a serine protease, instigates ferroptotic signaling by promoting the mobilization and subsequent esterification of AA *via* the regulation of ACSL4 (Tuo et al., 2022). Iron is also supposed to degrade H_2_O_2_ catalytically via the Fenton reaction to produce hydroxyl radicals, leading to the accumulation of ROS. 3) Amino acid axillary toxicity. Glutamate is a major excitatory neurotransmitter in the central nervous system, and its excessive release could cause neurotoxicity. After ischemia, the low expressions of glutamate transporter is observed and the time-dependent increasing of extracellular glutamate inhibits the cystine/glutamate antiporter (system Xc-), which cause the abnormal lipid peroxidation and ferroptosis (Yang et al., 2012; Krzyżanowska, et al., 2017). It has also been reported that HIF 1α decreases system Xc-expression, increases glutamate leakage in ischemic brain injury, and the activation of N-methyl D aspartate receptor (NMDAR) increases high extracellular glutamate concentrations and iron uptake in nerves (Krzyzanpwska et al., 2017; Hsieh et al., 2017; Cheah et al., 2006). The system Xc-is a heterodimeric amino acid transporter comprising a light chain xCT (SLC7A11) and a heavy chain 4F2hc (SLC3A2), which provides the raw meterials needed for GSH synthesis. Furthermore, the levels of SLC7A11 and GPX4 were found to be decreased significantly in MCAO rats compared to sham surgery (Lan et al., 2020), GPX4 inactivation has been attributed to GSH depletion during ischemia-reperfusion (Li et al., 2018), and lipid peroxide-mediated cell death in neurons (Seiler et al., 2008). In addition, tumor protein p53 (TP53, or p53) mediates (either promote or inhibit) ferroptosis *via* distinct mechanisms (Liu and Gu, 2021), such as p53 can increase the ferroptosis rate via inhibiting SLC7A11 expression (Jiang et al., 2015). Ferritin upregulation can prevent ferroptosis by mediating p53 and SLC7A11 expression in MCAO-induced hippocampal neuronal death (Chen et al., 2021). Additionally, the overexpression or knockdown of p53 significantly modulated GPX4 expression in RBMVECs exposed to the injury induced by OGD combined with hyperglycemic treatment (Chen et al., 2021).

**FIGURE 1:**
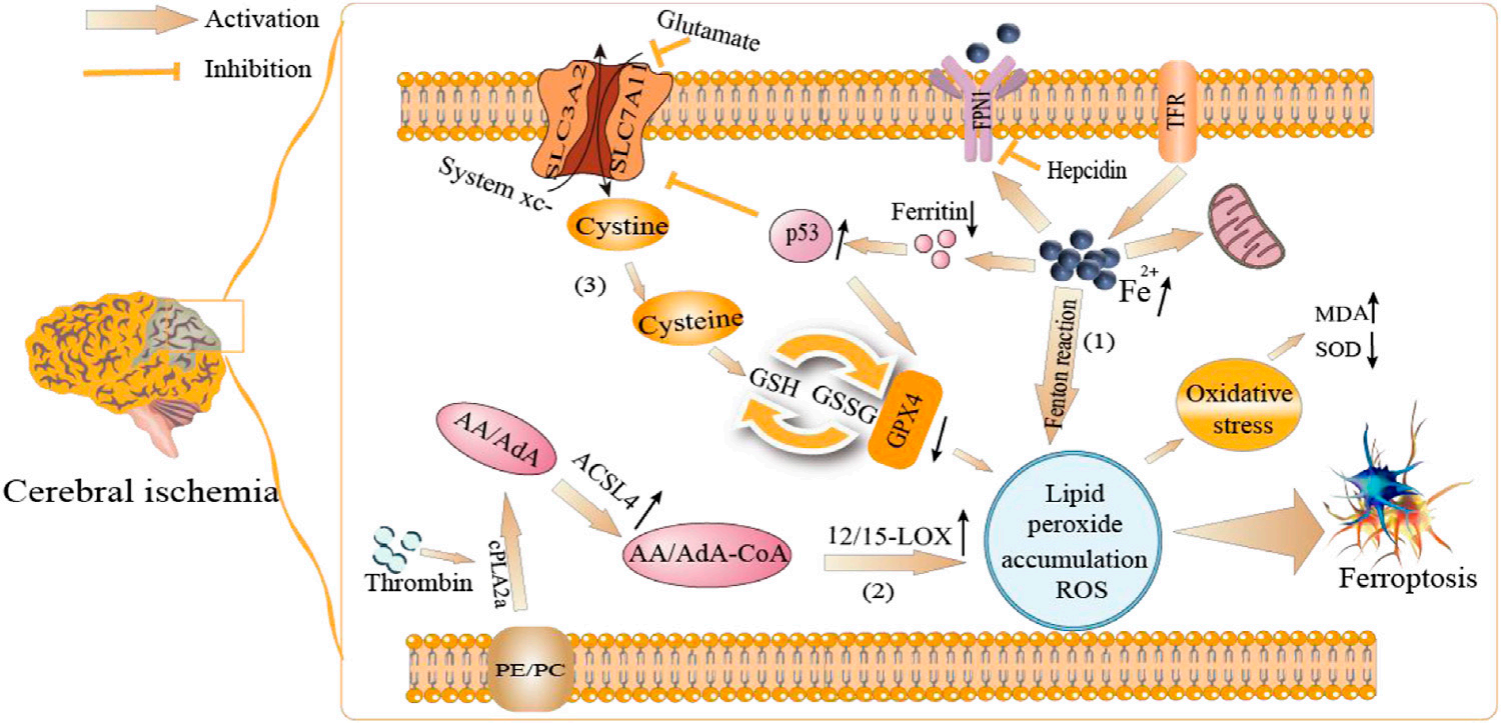
The current mechanisms of ferroptosis in cerebral ischemia is mainly attributed to: (1) Iron dyhomeostasis leading to iron overload and ferroptosis. (2) Metabolic imbalances of lipids aggravate accumulation of lipid peroxide and ROS. (3) Amino acid axillary toxicity causes system xc-is impaired, which inhibits cystine-glutamate exchange and decreases the generation of the antioxidant GSH and GPX4.

Owning to the description of above, ferroptosis may be induced during cerebral ischemia by pathological conditions such as iron imbalance, lipid peroxidation, aggregation of ROS, and axillary toxicity of amino acids. The specific mechanisms are shown in Figure 1.

## Traditional Chinese medicine targets interventional ferroptosis to alleviate cerebral ischemia

TCM has play a remarkable effect in the treatment of cancer and other serious diseases *via* ferroptosis signal pathway (Table 1). It is also important to explore the relationship between TCM and ferroptosis in the clinical treatment of cerebral ischemia and drug development.

**TABLE 1:** TCM targeted intervention ferroptosis to alleviate diseases.

Intervention on ferroptosis	TCM	Species, concentration	Experimental model	Biochemical indicators	Related diseases
inhibiting	Baicalein (Xie et al., 2016; Probst et al., 2017)	Roots of Scutellaria baicalensis/Scutellaria lateriflora	Erastin/RSL3-induced ferroptosis in pancreatic cancer cells/in acute lymphoblastic leukemia cells	ROS ↓、GPX4 ↑、Fe^2+^ ↓	Acute leukemia
	Puerarin (Liu et al., 2018)	Root of the Pueraria lobata (Willd.)Ohwi	Erastin/isoprenaline induced ferroptosis in H9c2 myocytes and in rats with aortic banding	ROS ↓、Fe^2+^ ↓	Heart failure
	Gastrodin (Jiang et al., 2020)	Rhizomes of the Gastrodia elata Blume	Glutamate-induced neurotoxicity in HT-22 cells	ROS ↓	Neurodegenerative diseases
	Xiaoyaosan (Jiao et al., 2021)	Radix Angelicae sinensis, Radix Paeoniae alba, Poria, Radix bupleuri, Rhizoma Atractylodis macrocephalae, Radix glycyrrhizae, Herba menthae, and Rhizoma Zingiberis recens, in a ratio of 3:3:3:3:3:1.5:1:1	Chronic unpredictable mild stress mices	GPX4 ↑、Fe^2+^ ↓	Depression
	Galangin (Guan et al., 2021)	Rhizomes of the alpinia officinarum hance	Bilateral common carotid artery ligation in gerbils	SLC7A11 ↑、GPX4 ↑	Cerebral ischemia
	Carthamin yellow (Guo et al., 2021)	Flower of the Carthamus tinctorius L	MCAO model rats	ROS ↓、Fe^2+^ ↓、ACSL4↓、GPX4 ↑、TFR1↓、FTH1↑	Cerebral ischemia
	Carvacrol (Guan et al., 2019)	The essential oil fractions of oregano and thyme	Bilateral common carotid artery ligation in gerbils	GPX4 ↑	Cerebral ischemia
	Tanshinone IIA (Xu ans Tang, 2019)	Root/rhizomes of the salvia miltiorrhiza bunge	Erastin-induced ferroptosis in HT22 cells	ROS ↓、Fe^2+^ ↓	Neurodegenerative diseases
	Naotaifang (Lan et al., 2020; He et al., 2020; Rao et al., 2021)	Astragali radix 40g, Chuanxiong Rhizoma10g, Pheretime 15g, Bombyx batryticetus15 g	MCAO model rats	ROS ↓、Fe^2+^ ↓、MDA ↓、SLC7A11 ↑、GPX4 ↑、GSH ↑	Ischemic stroke
	Ophiopogonin D (Lin et al., 2021)	Rhizomes of the ophiopogon japonicus (L. f.) Ker-Gawl	Ophiopogonin D’induced injury in H9c2 cell	Fe^2+^ ↑、ROS↑↓、 GSH ↑	Cardiomyopathy
	Electroacupuncture (Li et al., 2021)		MCAO model rats	ROS ↓、Fe^2+^ ↓、MDA ↓、SLC7A11 ↑、GPX4 ↑、	Cerebral ischemia
promoting	Artemisinin derivatives (Yu et al., 2015; Greenshields et al., 2017; Roh et al., 2017)	Sweet Wormwood/Artemisia annua L/artemisinin/	Erastin-induced ferroptosis in acute myeloid leukemia cells/mouse model of ovarian cancer/mouse tumor xenograft models	ROS ↑、GSH ↓、Fe^2+^ ↑	Acute myelogenous leukemia/ovarian cancer/head and neck cancer
	Piperlongumine (Yamaguchi et al., 2018)	Long pepper (Piper longum L.)	The human pancreatic cancer cell lines	ROS ↑、Fe^2+^ ↑	Pancreatic cancer
	Sijunzi Decoction (Wang et al., 2021)	Ginseng radix et rhizoma, Atractylodis rhizoma, poria	High-fat diet for mice	ROS ↑、SOD ↓、GSH ↓	Atherosclerosis
		9 g each, Glycyrrhizae radix et rhizoma 6 g			
	Ophiopogonin D′ (Lin et al., 2021)	Rhizomes of the ophiopogon japonicus (L. f.) Ker-Gawl	Ophiopogonin D’induced injury in H9c2 cell	Fe^2+^ ↑、ROS↑、GSH-Px ↑、 GSH↓	Cardiomyopathy
	Guizhi Fuling Capsule (Zhang et al., 2021)	Cinnamomum cassia Presl, Poria cocos (Schw.) Wolf (Poria), Paeonia suffruticosa Andr. (Moutan Cortex), Paeonia lactiflora Pall (Paeoniae Radix Alba), and Prunus persica (L.)	Estrogen to induce endometrial hyperplasia in mice	GPX4 ↓、MDA ↑	Endometrial hyperplasia

### Active ingredients in traditional chinese medicine

Galangin is a flavonol compound extracted mainly from alpinia officinarum hance and has multiple anti-tumor effects (Feng et al., 2022). Galangin reduced brain cell death in gerbils during cerebral ischemia-reperfusion (I/R), improved learning and memory ability apparently and increased SLC7A11 and GPX4 expression (Guan et al., 2021). Carthamin yellow is a flavonoid extracted from safflower, which is widely used for promoting blood circulation to remove blood stasis and alleviating pain in china (Lu et al., 2019). (Guo et al., 2020) found that carthamin yellow protected rats from ischemic injury by alleviating inflammation and the ferroptosis protection, also it was observed that the inhibition of Fe^2+^ and ROS accumulation and the reversion protein expression levels of ACSL4, TFR1, GPX4 and FTH1 in the brain after the treatment of carthamin yellow (Guan et al., 2021). Carvacrol is a monoterpenoid found in botanical drugs ang has multiple pharmacological effects (Silva et al., 2018). It has been proven to decrease cell death by increasing GPX4 expression and ferroptosis, realized neuroprotective effects of I/R injury in hippocampal neurons ultimately (Guan et al., 2019). Tanshinone IIA is the main fat-soluble component of Salvia.

Miltiorrhiza bunge (Guan et al., 2021), can inhibit ferroptosis in a cerebral ischemic cell model by regulating iron homeostasis, ROS accumulation and liposomal peroxidation (Xu et al., 2021).

### Traditional chinese medicine formula and acupunctur

Naotaifang, a TCM formula consisting of four botanical drugs, which can inhibit the development of ferroptosis by modulating the GPX4-lipid metabolic pathway in the MCAO model (He et al., 2020). In addition, Naotaifang extract can decrease the expression of TFR1 and DMT1 in MCAO rats by improving neurobehavioral scores, meanwhile the accumulation of ROS, MDA and iron were suppressed, and then regulates the expression of ferroptosis markers such as SLC7A11, GPX4, and GSH. It was confirmed that there was a protective effect against ferroptosis (Lan et al., 2020). Furthermore, Naotaifang inhibits neuronal iron absorption by suppressing TFR1 expression *via* the regulation of HSF1/HSPB1 signal pathway, a series of events such as the increasing expression of FTH1 and ferritin iron stores were detected, and then changes the steady-state balance of iron metabolism which would protect against ROS produced by the Fenton reaction and subsequent peroxidation of lipid-induced neuronal hyperferroptosis in ischemic stroke ultimately (Rao et al., 2021). The acupuncture has been used in China for more than 3,000 years, the modern study on the gave acupuncture stimulation to MCAO rats for seven consecutive days found that the acupuncture group decreased MDA and total iron levels compared to the MCAO group, while superoxide dismutase, GSH and GPX4 gene, FTH1, and FTH1 gene increased, TF, TF gene, TFR1, and TFR1 gene decreased in the MCAO rat model, suggesting that acupuncture exerts a protective effect in the MCAO rat model by modulating oxidative stress and iron-related proteins in the way of ferroptosis pathway. Experiment results have demonstrated that TCM prevents cerebral ischemia by regulating ferroptosis, but the lack of substantial evidence from human samples is the obstacle to our understanding of its potential clinical applications (Li et al., 2021).

## The crosstalk between ferroptosis and multiple cell death pathways

Cerebral ischemia, a complex cerebrovascular disorder, is regulated by multiple cell death pathways (Tuo et al., 2022). However, most mechanism studies tend to focus on a single cell death pathway. The individual cell death pathways do not appear to exist independently but have complex biological correlations and play a pivotal role in jointly maintaining the internal balance of various pathological conditions. Bold supposes that the blocking of PCD pathway could be a novel target for the prevention and treatment of various human diseases (Bedoui et al., 2020), and there are several results have been obtained as powerful evidence (Meng et al., 2021). Here, we focus on ferroptosis and discuss the relationship with other PCD to elucidate the crosstalk between multiple PCD pathways, it would be a valuable reference for the potential prevention and treatment of cerebral ischemia.

### Ferroptosis and apoptosis

The main pathway of neuronal injury after cerebral ischemia is apoptosis, as a classical cell death pathway, little is known about the potential interaction between ferroptosis and apoptosis in the disease of ischemic. Current studies often combine ferroptosis and apoptosis in anti-cancer therapy rather than cerebral ischemia (Fu et al., 2021; Ye et al., 2021; Zhang et al., 2021). For example, Meng et al. proposed a high-performance pyrite nanoenzyme can kill tumor cells in an anti-apoptotic manner by inducing ferroptosis (Meng et al., 2021); Bao et al. not only verified the self-made nanolongans can carry multiple targeted drugs to the goal sites, but also demonstrated that ferroptosis and apoptosis were both suppression when using a combination of co-targeted anti-cancer therapies (Bao et al., 2019). Logie et al. (2021) showed that cell death by apoptosis and ferroptosis induce different changes of kinase signaling in multiple myeloma cells (Logie et al., 2021). On the other hand, in a related study, apoptosis-dependent ferroptosis was found have occured in leukemia cells (Wang et al., 2020), suggesting a possible interdependence of the two PCD. Ferroptosis inducers also is sensitive to apoptosis by increasing the expression of PUMA, an apoptosis-inducing molecule (Hong et al., 2017). Furthermore, the combination of erastin (ferroptosis inducer) and TRAIL (apoptosis induce ligand) disrupted mitochondrial membrane potential (ΔΨm) effectively, subsequently promoting caspase activation (Lee et al., 2020). In addition, studies have shown that apoptosis/ferroptosis is ROS-dependent (Zhang et al., 2021), and they can be regulated by regulating p53 (Mao et al., 2018; Li et al., 2020). These findings indicate that a combination of ferroptosis and apoptosis is feasible, but the applicability and specific mechanisms of apoptosis and ferroptosis in the pathway of neuronal injury after cerebral ischemia need to be further investigated.

### Ferroptosis and autophagy

Autophagy is a dynamic process that depends on the formation and maturation of specific membrane structures, playing a dual role in the regulation of cerebral ischemia. With the intensive researches, ferroptosis was suggested as a type of autophagy-dependent cell death pathway (Zhou et al., 2020). Autophagy is also thought to contribute to iron homeostasis and ROS production, ultimately causing ferroptosis in the cell experiments (Gao et al., 2016). Furthermore, regulatory factors of ferroptosis were found to be transported to lysosomes and exert their functions through autophagy (Zhou et al., 2019). Their molecular mechanisms include NCOA4 (nuclear receptor coactivator 4)-dependent degradation of ferritin by ferritinophagy (Hou et al., 2016) and the inhibition of SLC7A11 activity by the formation of BECN1-SLC7A11 protein complex (Kang et al., 2018). Furthermore, RAB7A, SQSTM1, HSP90, and p53 are also thought to induce ferroptosis by mediating different types of autophagy (Jiao et al., 2021; Liu and Gu, 2022). Recently, it has become clearly that the activation of ferroptosis depends on the induction of autophagy (Kang and Tang., 2017; Torii et al., 2016). For example, the RNA-binding protein ZFP36/TTP is known to protect against ferroptosis by regulating autophagy signaling pathways in hepatic astrocytes (Zhang et al., 2020). However, autophagy markers are not closely related to ferroptosis regulators in the early induction of ferroptosis (Li et al., 2021). Coincidentally, the inhibition of autophagy increases the susceptibility of glioblastoma stem cells by igniting ferroptosis (Buccarelli et al., 2018). In addition, cell death is caused by ferroptosis and autophagy after the treatment of breast cancer cells with siramesine and lapatinib independently (Ma et al., 2017). These complex results may be caused by the differences of disease types and experimental models. There is studies indicate that autophagy and ferroptosis may mediate their interaction to control cerebral ischemia (Liu et al., 2020), but the relationship between the two processes remains a cluster of unknowns for the further research.

### Ferroptosis and pyroptosis and necroptosis

During cerebral ischemia, neuronal pyroptosis and necroptosis are extensively affected (Xu et al., 2018; Dong et al., 2018). Pyroptosis is thought to be executed by receptor-interacting protein kinase 3 (RIPK3) and a pseudokinase, mixed lineage kinase domain-like protein (MLKL). Previous studies have shown that excessive ROS levels induce gasdamine D (GSDMD)-mediated pyroptosis (Wang et al., 2020). When cecum ligation and puncture mice are treated with Fer-1, pyroapoptotic proteins are simultaneously regulated by GPX4 and PTGS2 (Cao et al., 2022). Furthermore, GPX4 can reduce lipid peroxidation and negatively regulate macrophage pyroapoptosis and polymicrobial sepsis in mice (Kang et al., 2018). Xu et al. also achieved dual induction of ferroptosis/pyroptosis in cancer therapy by aggregation of transferrin and ROS (Xu et al., 2021). Some findings confirmed that pyroptosis and ferroptosis occur in liver injury caused by microplastics (Mu et al., 2022), also found that NLRP3 inflammasome-dominated pyroptosis is involved in the induction of ferroptosis in type 2 diabetes (Chen et al., 2022). Conversely, (Wang et al., 2021), found that the histone deacetylase inhibitor xinostat induced cellular pyroptosis *via* the caspase-1-related pathway and ferroptosis *via* the GPX4-related pathway in TSCC cells (Wang et al., 2021). Wang J et al. found that pyroptosis and ferroptosis occur in early and late stages of chronic heart failure, respectively (Wang et al., 2020). These results add complexity and uncertainty to the interpretation of the crosstalk between pyroptosis and ferroptosis.

Necroptosis is a caspase-independent mode of cell death. A growing body of evidence suggests that both ferroptosis and necroptosis are involved. *In vivo* and *in vitro* model of neuronal cell death after stroke hemorrhage, the involvement of ferroptosis and necroptosis has been confirmed (Zille et al., 2017). Human neuroblastoma cell lines, a widely used model of Parkinson’s disease, treating with 1-methyl-4-phenylpyridium (MPP+), can induce necroptotic and nonapoptotic cell death, but the progress was been restrained by both treatment of necrostatin-1 and ferrostatin-1 (Ito et al., 2017). Proteomic analysis of the hippocampus revealed the activation of necroptosis and ferroptosis in a mouse model of chronic unpredictable mild stress-induced depression (Cao et al., 2021). The inhibition of mitochondrial complex I induced mitophagy-dependent ROS accumulation via depolarization of mitochondrial membrane potential, leading to the activation of necroptosis along with ferroptosis in melanoma cells (Efferth,2017). And there are crosstalk between ferroptosis and necroptosis were observed apparently in tumor cells and H/R-treated H9c2 cell lines when treating with some agonists. (Feng et al., 2022; Tu et al., 2021). More importantly, exploring the crosstalk between ferroptosis and necroptosis is thought to be a novel therapeutic strategy for cerebral ischemia (Zhou et al., 2021). Mechanistically, GSH degradation by CHAC1 promotes cystine starvation-induced necroptosis and ferroptosis in human triple-negative breast cancer cells *via* the GCN2-eIF2α-ATF4 pathway (Chen et al., 2017). Also HSP90 defines the regulatory intersection of necroptosis and ferroptosis (Wu et al., 2019). Furthermore, iron excess, one of the mechanisms of ferroptosis, leads to the opening of the mitochondrial permeability transition pore (MPTP), which exacerbates RIP1 phosphorylation and contributes to necroptosis (Zhou et al., 2021).

Crosstalk between multiple PCD is a new target for new drug discovery, and a vast amount of research has shown that the regulation of multiple PCD simultaneously would be a promising strategy for the treatment of disease. Artesunate (Efferth, 2017; Efferth, 2017), a widely prescribed antimalarial drug, has been reported to induce necroptosis and ferroptosis in tumor cells. Saudi Arabia chloride (Mbaveng et al., 2021) also has been reported to induce apoptosis, ferroptosis, necroptosis, and autophagy in malignant tumors, including refractory cancers. Palladium pyrithione complex (Yang et al., 2021) as a kind of broad-spectrum proteasomal deubiquitinase inhibitor, have been reported to inhibit tumor growth by activating caspase-dependent apoptosis and GPX4 degradation-dependent ferroptosis. However, these multifunctional agents are currently under-studied and require further research and development.

## Important potential applications of traditional chinese medicine in cerebral ischemia

**FIGURE 2:**
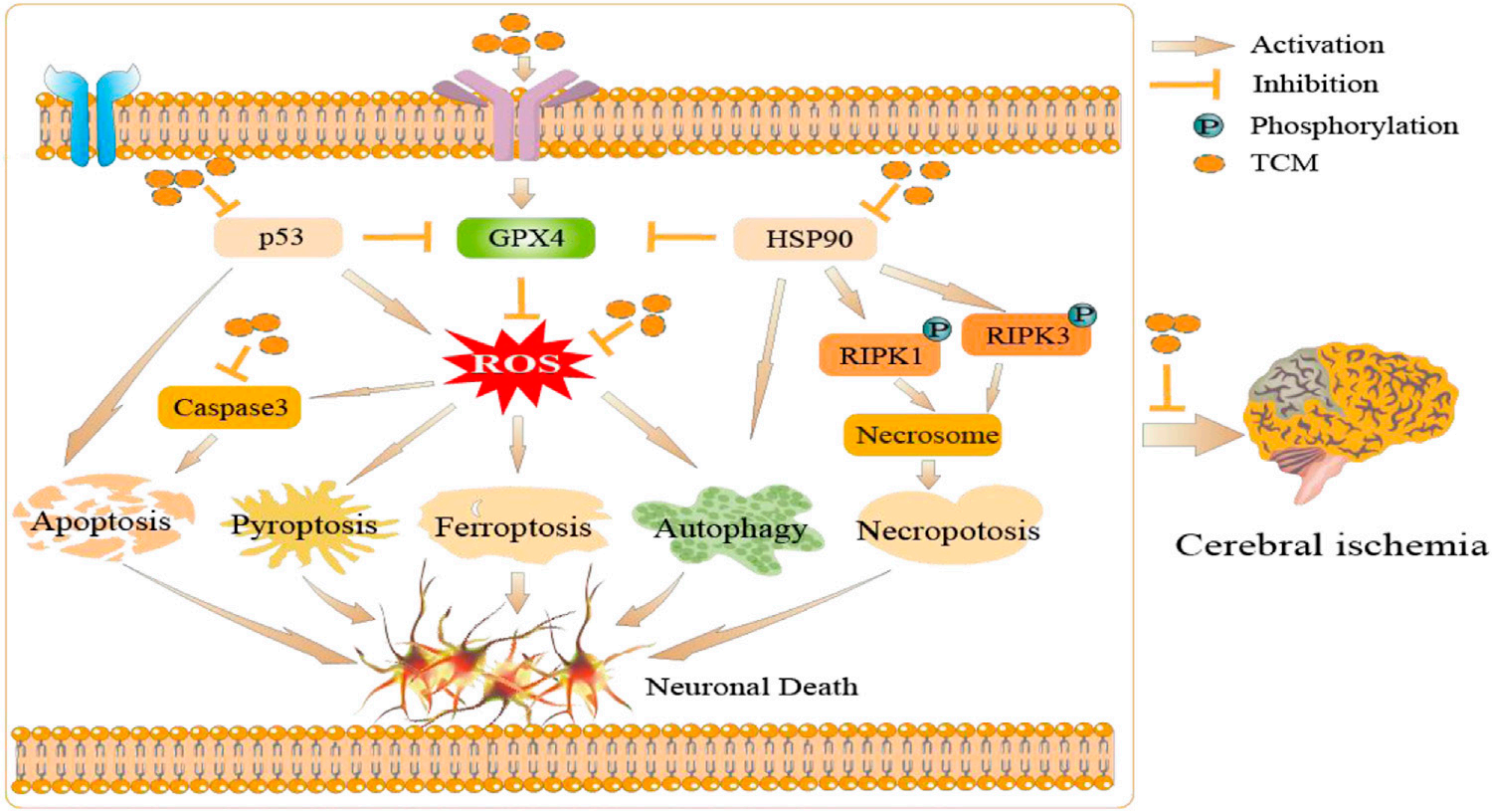
The potential applications of TCM in cerebral ischemia: TCM can regulate a variety of cell death pathways through ROS, HSP90, p53, and other molecular mechanisms, and thus play an anti-cerebral ischemia role.

TCM has unique clinical advantages due to its multiple targets, multiple effects, and multiple pathways (Chen, 2012). Importantly, an increasing number of studies suggest a modulatory effect of TCM on various cell death pathway in experimental models related to cerebral ischemia. Total Saponins of Panax Notoginseng, the main component extracted from Panax Notoginseng, has neuroprotective effects against focal ischemia. It has been reported that it may be associated with the inhibition of apoptosis (Li et al., 2009). Puerariae radix, the familiar kudzu root, is used to protect the brain from cerebral ischemic injury via the inhibition of astrocyte apoptosis (Wang et al., 2014). Curcumin and eugenol, polyphenolic compounds extracted from Curcuma longa, have been noted as being effective against cerebral ischemia-reperfusion by alleviating inflammation and mediating autophagy. (Huang et al., 2018; Sun et al., 2020). Dendrobium alkaloids have been reported to inhibit neuronal cell death by pyroptosis and promote neuronal function after cerebral ischemia-reperfusion injury *in vivo* and *in vitro* models (Liu et al., 2020). Tongxinluo, a common TCM prescription, has inhibitory effects on pyroptosis and amyloid-β peptide accumulation after rat cerebral ischemia-reperfusion (Wang et al., 2021). Huwentoxin also maintained the original morphology of mitochondria, decreased the expression of tumor necrosis factor, and protected neurons in rats with cerebral ischemia-reperfusion injury (Wang et al., 2014). In addition, a previous study by our research group have found that Buyang Huanwu Decoction and its similar formulations play the neuroprotective effects by regulating apoptosis and autophagy dynamically in oxidative stress model cells (Liu et al., 2020), and potential regulation of ferroptosis signal pathway with Buyang Huanwu Decoction were explored for the treatment of cerebral ischemia by network pharmacology. (Zhao et al., 2021). Yan She et al. also found that the glycoside of Buyang Huanwu Decoction has a protective effect against pyroptosis after cerebral ischemia-reperfusion injury in rats (She et al., 2019), suggesting that Buyang Huanwu Decoction may regulate apoptosis, autophagy, ferroptosis and pyroptosis during cerebral ischemia. Based on the above, we suppose that TCM can play a neuroprotective effect by regulating various cell death pathways through various molecular mechanisms, which is consistent with the characteristics of multi-target of TCM, and it is expected to reveal the important potential applications of TCM for cerebral ischemia (Figure 2).

## Discussion and prospects

Studies have suggested that various types of PCD are involved in cerebral ischemia, among which the research and development of drugs targeting ferroptosis has attracted great attention. At present, it remains to be clarified whether compounds targeting ferroptosis regulators have high specificity and adverse reactions in preclinical and clinical environments, while TCM has the advantages of higher clinical value and less side effects due to its clinical origin. However, TCM also has disadvantages such unclear pharmacodynamic substance basis and imperfect functional mechanism. Besides, there are many problems about mechanism to be resolved. For instance, studies have shown that ferroptosis can protect cells from oxidative stress (Fearnhead et al., 2017), suggesting that ferroptosis may also play a dual role in cerebral ischemia. Meanwhile, ferroptosis may also interact with other cell death during cerebral ischemia. Consequently, the resolution of these questions is expected to provide essential directions and targets for the prevention and treatment of cerebral ischemia.

Many existing studies have used the combined therapies to prevent and control diseases. (Zheng et al. (2017)). designed organometallic networks encapsulating p53 plasmids to kill cancer cells by ferroptosis/apoptosis hybridization (Zhang et al., 2021). Weier Bao et al. created nanorongeons capable of carrying multiple targeted drugs for anticancer therapy, the results showed that it can simultaneously intervene ferroptosis and apoptosis (Bao et al., 2019). Although the prevention and treatment of cerebral ischemia by combining various cell death pathways presents exciting possibilities, there are still many problems in current researches. For example, all types of cell death pathways are limited by the development of corresponding inducers and inhibitors. More complicatedly, the dynamics sequence of various cell death events and the relationship between the dual role of cell death *in vivo* and pathological processes are still unclear. Various problems need to be further studied by comparing the expression of different cell death forms at different times and grouping studies with rational use of various exclusive inhibitors, etc. This paper comprehensively explores the potential pathophysiology of cerebral ischemia, with emphasis on the potential application of TCM to the neuronal cell death pathway, which is helpful to futher promote and improve the relevant research and development of TCM in the treatment of cerebral ischemia.

## Abbreviations

TCM, Traditional Chinese medicine; PCD, programmed cell death; TCM, traditional Chinese medicines; GPX4, glutathione peroxidase 4; ROS, reactive oxygen species; GSH, glutathione; MCAO, middle cerebral artery occlusion; TFR, transferrin receptor; FPN1, ferroportin 1; ACSL4, acyl-coa synthetase long-chain family member 4; 12/15-LOX, 12/15-lipoxygenase; PUFAs, polyunsaturated fatty acids; AA, arachidonic acid, AdA, adrenal acid; system Xc-, cystine/glutamate antiporter; NMDAR, N-methyl D aspartate receptor; SLC7A11, suppressing solute carrier 7A11; SLC3A2, solute carrier 3A2; p53, Tumor protein p53; RIPK3, receptor-interacting protein kinase 3.
